# An Alternate STAT6-Independent Pathway Promotes Eosinophil Influx into Blood during Allergic Airway Inflammation

**DOI:** 10.1371/journal.pone.0017766

**Published:** 2011-03-15

**Authors:** Wan Wang, Philip M. Hansbro, Paul S. Foster, Ming Yang

**Affiliations:** Centre for Asthma and Respiratory Disease, School of Biomedical Sciences and Pharmacy, Faculty of Health, University of Newcastle and Hunter Medical Research Institute, Callaghan, New South Wales, Australia; McMaster University, Canada

## Abstract

**Background:**

Enhanced eosinophil responses have critical roles in the development of allergic diseases. IL-5 regulates the maturation, migration and survival of eosinophils, and IL-5 and eotaxins mediate the trafficking and activation of eosinophils in inflamed tissues. CD4^+^ Th2 cells are the main producers of IL-5 and other cells such as NK also release this cytokine. Although multiple signalling pathways may be involved, STAT6 critically regulates the differentiation and cytokine production of Th2 cells and the expression of eotaxins. Nevertheless, the mechanisms that mediate different parts of the eosinophilic inflammatory process in different tissues in allergic airway diseases remain unclear. Furthermore, the mechanisms at play may vary depending on the context of inflammation and microenvironment of the involved tissues.

**Methodology/Principal Findings:**

We employed a model of allergic airway disease in wild type and STAT6-deficient mice to explore the roles of STAT6 and IL-5 in the development of eosinophilic inflammation in this context. Quantitative PCR and ELISA were used to examine IL-5, eotaxins levels in serum and lungs. Eosinophils in lung, peripheral blood and bone marrow were characterized by morphological properties. CD4^+^ T cell and NK cells were identified by flow cytometry. Antibodies were used to deplete CD4^+^ and NK cells. We showed that STAT6 is indispensible for eosinophilic lung inflammation and the induction of eotaxin-1 and -2 during allergic airway inflammation. In the absence of these chemokines eosinophils are not attracted into lung and accumulate in peripheral blood. We also demonstrate the existence of an alternate STAT6-independent pathway of IL-5 production by CD4^+^ and NK cells that mediates the development of eosinophils in bone marrow and their subsequent movement into the circulation.

**Conclusions:**

These results suggest that different points of eosinophilic inflammatory processes in allergic airway disease may be differentially regulated by the activation of STAT6-dependent and -independent pathways.

## Introduction

Eosinophilic inflammation is a hallmark feature of allergic diseases of the lung (asthma), gastrointestinal tract (allergic eosinophilic gastroenteritis), skin (eczema), other systemic diseases (idiopathic hypereosinophilic syndrome and eosinophilic pneumonia) and parasitic helminth infection [1]. Eosinophils play an important pathogenetic role in the processes that lead to the precipitation of these diseases by releasing a wide range of cytotoxic products and proinflammatory factors [1], [2]. A substantial body of research has elucidated the major molecular processes that regulate the development of eosinophilic inflammation. Eosinophils differentiate in the bone marrow from pluripotent stem cells and IL-3, IL-5 and GM-CSF are particularly important factors that promote their development [1], [3].

IL-5 is the most important factor that regulates the expansion, growth and survival of eosinophils although it is dispensable for eosinophil development under homeostatic conditions [4]. This cytokine also directly promotes allergic airway disease by mediating eosinophilic inflammation [5]. Indeed many diseases that have accompanying eosinophilic inflammation are often associated with increased expression of IL-5 [6]. Importantly, this cytokine provides a critical signal for the eosinophilic response in bone marrow and the subsequent release of this cell into peripheral blood in response to inflammatory stimulation [5], [7]. Mice deficient in IL-5 have reduced numbers of eosinophils in peripheral blood and bone marrow and mice over-expressing IL-5 have increased infiltrations of eosinophils into many tissues (e.g. spleen, bone marrow, lung and lymph nodes) [4], [8]. Nevertheless, the cellular and molecular mechanisms that mediate the production of IL-5 and the subsequent development of eosinophilic responses have not been fully elucidated.

Once eosinophils are produced specific chemotactic factors, namely the chemokines eotaxin-1, -2 and -3, cooperate with IL-5 to critically regulate their migration and activation during allergic inflammation [1]. These chemokines possess common biologic functions but regulate different phases of eosinophil recruitment during allergic inflammation in humans, although only eotaxin-1 and -2 have been identified in mice [1]. Eotaxins also induce rapid and transient actin polymerization, upregulate integrin function, and modulate respiratory burst in eosinophils [1].

Many immune cells, in particular CD4^+^ T-helper type 2 lymphocytes (Th2 cells), CD8^+^ T cells, and NK cells but also mast cells and eosinophils produce IL-5. Of these cells, Th2 cells are the predominant source of IL-5 during allergic responses [9]–[11]. NK cells have also been demonstrated to secrete IL-5 and actively regulate the development of eosinophilic inflammation in human and animal studies [9], [12]. Although NK cells are well known to critically regulate both Th1 and Th2 responses [13], their roles in the regulation of eosinophilic responses in bone marrow during allergic inflammation remains incompletely understood.

Clinical and experimental investigations have demonstrated the obligatory role of Th2 cells in the pathogenesis of eosinophilic inflammation and allergic disorders [14]–[16]. STAT6 is a critical factor for efficient Th2 polarization [17], [18] and the expression of eotaxins [19]–[21]. Indeed, STAT6-deficient mice do not develop AHR or eosinophilic airway inflammation in mouse models of allergic airway disease [22]–[24]. By contrast, in other systems, STAT6 is not required for tissue eosinophilia [25], [26]. Furthermore, there is evidence that STAT6-independent IL-5 production is involved in eosinophilic inflammation of the intestine during *Nippostrongylus brasilliensis* infection in mice [27]. The role of STAT6 in different parts of the eosinophilic inflammatory processes in allergic airway disease is not understood. Furthermore, the contribution and roles of STAT6 in mediating the production of IL-5 and eotaxins and in the development of eosinophils in the bone marrow, their release into blood and in the progression of eosinophilic inflammation remains poorly characterized.

In this study we assessed the role of STAT6 in the development of eosinophilic inflammation in a mouse model of allergic airways inflammation using wild type (WT) and STAT6-deficient mice. We also determined the roles of IL-5, eotaxins, CD4^+^ and CD8^+^ T cells and NK cells in the development of STAT6-independent eosinophilic inflammation.

## Materials and Methods

### Animals

Specific pathogen free WT and STAT6-deficient BALB/c mice (male and female, 6–8 weeks) were obtained from the University of Newcastle and Australian National University. STAT6-deficient mice were backcrossed for 12 generations onto the BALB/c background. All experiments were performed with approval from the animal ethics committees of The University of Newcastle (ID 899 and 974) and the Australian National University (The early part of experiments was conducted at ANU and ANU ID was expired and not archived).

### Induction of allergic airways inflammation

Mice were sensitized at 6–8 wk of age by i.p. injection (day 0) with 50 µg of ovalbumin (OVA) (fraction V, Sigma, St Louis, MO, USA) admixed with 1 mg Alhydrogel (Reheis Inc., Berkeley Heights, NJ, USA) in 200 µl of 0.9% sterile saline on day 0 and 12. Non-sensitized mice were injected with 1 mg Alhydrogel in 200 µl of 0.9% sterile saline. On days 24, 26, 28 and 30, all groups of mice were challenged with aerosolized OVA (10 mg/ml in 0.9% saline) for 3×30 minutes with 30 minutes break using an ultrasonic nebuliser. On day 23, 25, and 27, 29 and 31, eosinophils as a percentage of leukocytes in peripheral blood were assessed. On day 31 inflammatory responses in bronchoalveolar lavage, airway sections and lung tissue, and eosinophil numbers in the bone marrow were assessed as previously described [28]–[30].

### Bronchoalveolar lavage and histopathology

Bronchoalveaolar lavage was collected, cells isolated and stained and differential inflammatory cell counts performed as previously described [30]. Lungs were fixed in 10% phosphate-buffered formalin, sectioned, and stained with carbol chromotrope and eosinophils enumerated as previously described [31].

### ELISA Analysis

Blood was collected by heart bleed and cell-free serum prepared. IL-5, eotaxin-1 and -2 were determined by ELISA cytokine according to the instructions of the manufacturer (BD Pharmingen, San Diego, CA, USA) [30].

### Quantitative PCR

The method for quantitative PCR has been described in detail previously [32]. Briefly, RNA was prepared from cells or tissue using the TRIzol RNA isolation buffer following the protocol of the manufacturer (Invitrogen Life Technologies). cDNA was synthesized by an oligo(dT) primed reverse transcriptase reaction using 0.5 µg of RNA from each sample. Quantitative PCR was performed using an ABI PRISM 7000 Sequence Detection System (Applied Biosystems) using the following primers: murine IL-5 (forward, CTCTGTTGACAAGCAATGAGACG and reverse, TCTTCAGTATGTCTAGCCCCTG); eotaxin-1 (forward, CCCAACACACTACTGAAGAGCTACAA, and reverse, TTTGCCCAACCTGGTCTTG); eotaxin-2 (forward, TAGCCTGCGCGTGTTGCATCTTCC, and reverse, TAAACCTCGGTGCTATTGCCACGG); and GAPDH (forward, CAGGTTGTCTCCTGCGACTT, and reverse, CCCTGTTGCTGTAGCCGTA). SYBR-green was used to detect changes in amplicon levels with each sequential amplification cycle. The fluorescence intensity was normalized to the rhodamine derivative ROX as a passive reference label, which was present in the buffer solution. The level of mRNA, normalized to GAPDH, was calculated as fold change.

### Flow cytometry

Bone marrow was isolated as previously described [33]. Samples were dissociated into single cell suspensions, and depleted of erythrocytes using 0.86% (w/v) ammonium chloride. Cells were washed and stained for surface marker expression using fluorescent monoclonal antibodies. Anti-CD3, anti-CD4 and anti-CD8 (Biolegend, San Diego, CA, USA) were used to detect CD4^+^ or CD8^+^ T cells and anti-CD3, anti-CD49b and anti-FcεRII (Biolegend) were used to detect NK cells (CD3^−^CD49b^+^FcεRII^−^). All samples were analysed using a FACSCanto flow cytometer and associated software (BD Bioscience, San Jose, CA, USA).

### Depletion of IL-5, CD4^+^ and CD8^+^ T cells and NK cells

Mice were injected i.p. with anti-IL-5 (200 ug, TFK5, rat anti-mouse monoclonal IgG1, ATCC, Manassas, VA, USA) or isotype control (rat IgG1) monoclonal antibody (mAb) on days 22, 26 and 30 during the OVA challenge as previously described [34]. Depletion of IL-5 was confirmed in serum samples by ELISA.

CD4^+^ or CD8^+^ cells were depleted by i.p. injection with 500 µg anti-CD4 (Clone GK1.5, rat anti-mouse monoclonal IgG_2b_) [35] or anti-CD8 (Clone YTS169.4, rat anti-mouse monoclonal IgG_2b_) mAbs [36] or the corresponding isotype control (rat IgG_2b_), on days 22, 26 and 30. Depletion of CD4^+^ or CD8^+^ T cells was confirmed in spleens by FACS analysis.

NK cells were depleted by i.v. injection with 50 µl anti-ASIALO GM1 polyclonal antibody (Wako Chemicals, Osaka, Japan) or rabbit serum on day 22, 26 and 30, according to manufacturer's instructions as previously described [37]. Depletion of NK cells was determined in spleens by FACS analysis. In some experiments, both CD4^+^ cells and NK cells were depleted by combined treatment with anti-CD4 and anti-ASIALO GM1 antibodies.

### Data analysis

An initial one-way analysis of variance (or a Kruskal-Wallis test for non-parametric data) was followed by appropriate comparisons to test for differences between means of groups. Values are reported as the mean ± SEM for each experimental group. The number of mice in each group ranged from 8–12. Differences in means were considered significant if P<0.05.

## Results

### STAT6 is required for the influx of eosinophils into the lungs during allergic airway inflammation

**Figure 1:**
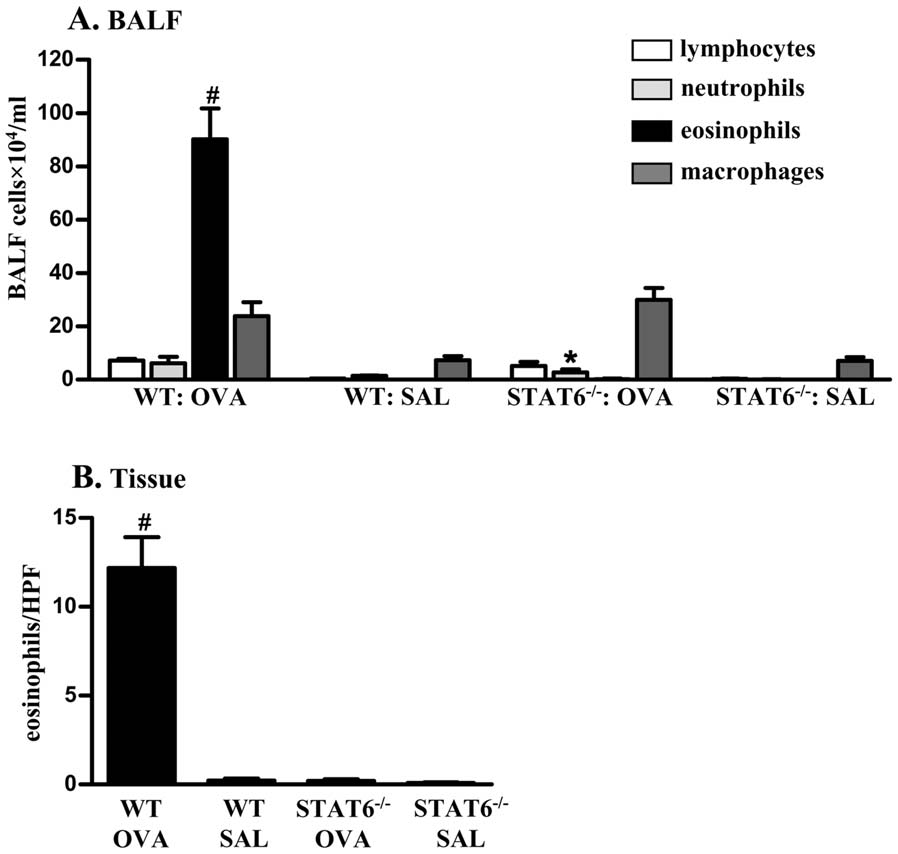
Characterization of eosinophilic inflammation in allergic lungs. OVA/OVA treated WT mice had significantly increased numbers of eosinophils in (A) BALF and (B) peribronchial and perivascular regions of the lung, compared to SAL/OVA treated controls. The levels of BALF neutrophil in OVA/OVA treated STAT6-deficient mice were significantly higher than that in SAL/OVA WT control but less than that in OVA/OVA treated WT mice. No significant infiltration of eosinophils was observed in OVA/OVA treated STAT6-deficient mice, which were at the same levels as SAL/OVA treated WT mice. #P<0.05 compared to SAL/OVA treated WT, *P<0.05 compared to OVA/OVA treated WT mice.

We first investigated the role of STAT6 in eosinophil accumulation in the lung. WT and STAT6-deficient mice were sensitized and challenged with OVA/OVA or SAL/OVA and leukocyte numbers were determined in the BALF. Pronounced infiltrations of inflammatory cells were detected in the BALF of OVA/OVA treated WT mice, compared to SAL/OVA treated controls (Figure 1A). In particular, the levels of eosinophils were substantially increased (20 fold). By contrast, the numbers of eosinophils in OVA/OVA treated STAT6-deficient mice were not significantly increased compared to SAL/OVA treated controls, and were similar to those in SAL/OVA treated WT mice. Neutrophils in OVA/OVA treated STAT6-deficient mice were increased compared to SAL/OVA treated controls but were significantly decreased compared to OVA/OVA treated WT mice. The infiltrations of lymphocytes and macrophages were increased and were similar in both OVA/OVA treated WT and STAT6-deficient groups.

Histological examination of lung tissue showed that a significant infiltration of eosinophils occurred into the peribronchial and perivascular regions of OVA/OVA treated WT mice compared to SAL/OVA treated controls (Figure 1B). Consistent with the observations in BALF, eosinophil accumulation in lung tissue was absent in OVA/OVA treated STAT6-deficient mice, and was similar to that in SAL/OVA treated WT mice. These results demonstrate that STAT6 is required for eosinophilic inflammation in the lung.

### Deficiency in STAT6 results in enhanced eosinophil accumulation in peripheral blood but impaired development in bone marrow

**Figure 2:**
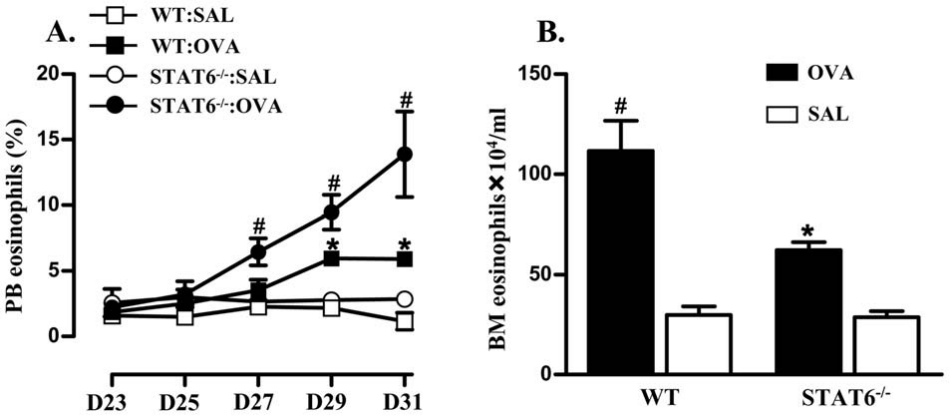
Eosinophil levels in peripheral blood and bone marrow. OVA/OVA treatment of WT and STAT6-deficient mice significantly increased (A) levels of eosinophils as percentages of leukocytes in peripheral blood and (B) numbers of eosinophils in bone marrow compared to SAL/OVA treated controls. OVA/OVA treated STAT6-deficient mice had a greater percentage of eosinophils in peripheral blood but significantly fewer eosinophils in the bone marrow than OVA/OVA treated WT controls. #P<0.05 compared to other groups. *P<0.05 compared to SAL/OVA treated controls.

To determine the point of the inflammatory pathways where eosinophil influx in the lung was disrupted in STAT6-deficient mice, eosinophil levels in the blood and bone marrow were assessed. The percentages of eosinophils in peripheral blood of OVA/OVA treated WT and STAT6-deficient mice were significantly greater than in respective SAL/OVA treated controls (Figure 2A). In the absence of STAT6, there was an increase in the accumulation of eosinophils in peripheral blood in response to OVA/OVA treatment compared to WT mice. No increase of eosinophil percentages in the blood was observed in SAL/OVA treated WT or STAT6-deficient mice.

OVA/OVA treatment of WT and STAT6-deficient mice resulted in increased numbers of eosinophil in bone marrow compared to the respective SAL/OVA controls (Figure 2B). The number of eosinophils in the bone marrow of OVA/OVA treated STAT6-deficient mice was significantly lower than in OVA/OVA treated WT mice. Eosinophils numbers in SAL/OVA treated WT and STAT6-deficient mice were not significantly different.

### STAT6 contributes to IL-5 production and is required for the expression of eotaxins

**Figure 3:**
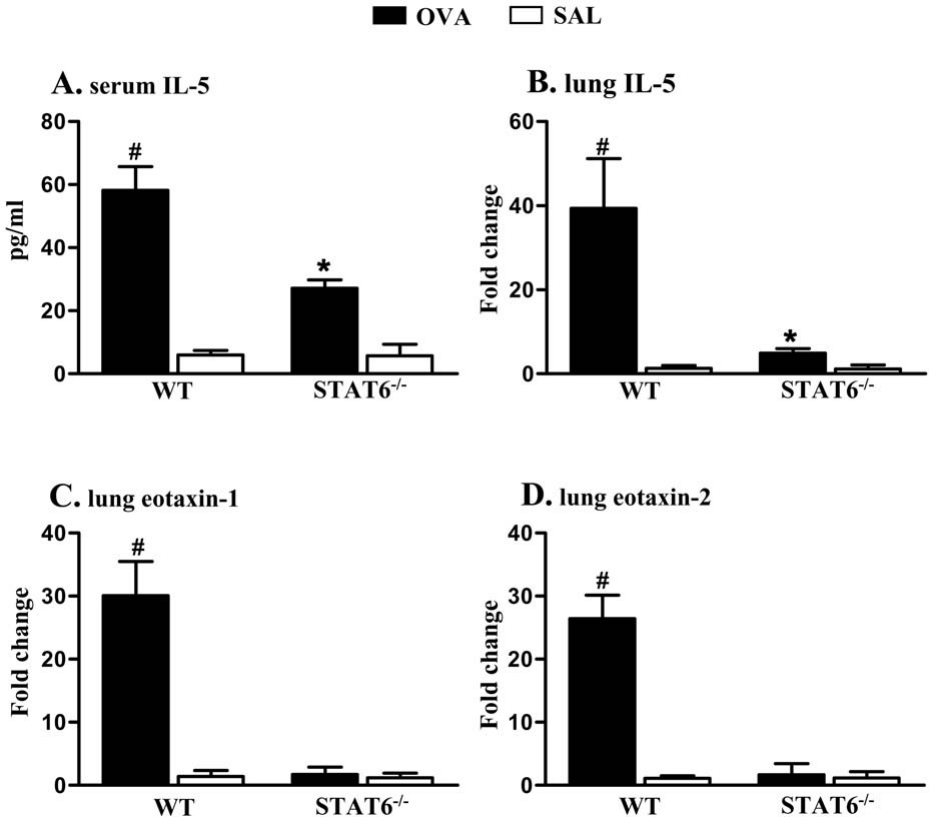
The levels of IL-5 in serum and expression of eotaxin-1 and -2 mRNA in lungs. OVA/OVA treated WT mice had significantly increased levels of (A) serum IL-5 and mRNA encoding (B) IL-5, (C) eotaxin-1 and (D) eotaxin-2 in lung tissue compared to SAL/OVA treated controls. OVA/OVA treated STAT6-deficient mice also had significantly increased levels of IL-5 in serum and lung but these levels were lower than OVA/OVA treated WT mice. There was no increase the expression of eotaxin-1 and -2 in OVA/OVA treated STAT-6 deficient or SAL/OVA treated controls. #P<0.05 compared to other groups. *P<0.05 compared to respective SAL/OVA treated control.

IL-5 and eotaxins are critical regulators of the expansion and chemotaxis of eosinophils [38]. Therefore, we assessed whether the lack of eosinophilic inflammation in the absence of STAT6 resulted from reduced IL-5 or eotaxin responses. OVA/OVA treatment of WT mice increased the levels of IL-5 in serum compared to SAL/OVA controls (Figure 3A). OVA/OVA treatment of STAT6-deficient mice also increased IL-5 levels but to a lower level than in WT mice. No increased levels of eotaxin-1 and -2 were detected in the serum of all groups (unpublished data). OVA/OVA treatment of WT mice profoundly increased the mRNA expression of IL-5, eotaxin-1 and -2 in lung tissues (Figure 3B–D). Levels of IL-5 in the lung of OVA/OVA treated STAT6-deficient mice were markedly decreased compared to OVA/OVA treated WT mice but were still significantly higher than that of respective SAL/OVA group (Figure 3B). However, no increase of eotaxin-1 and -2 was observed in OVA/OVA treated STAT6-deficient mice or SAL/OVA treated controls. These results indicate that STAT6 does contribute to IL-5 production and eosinophil accumulation in the lung tissue. However, IL-5 production and eosinophil development and influx into peripheral blood may also occur *via* a STAT6-independent pathway. These results also suggest that STAT6-dependent production of eotaxins is required for the influx of eosinophils into lung tissue.

### STAT6-independent IL-5 production contributes to eosinophil accumulation in peripheral blood and development and expansion in bone marrow

**Figure 4:**
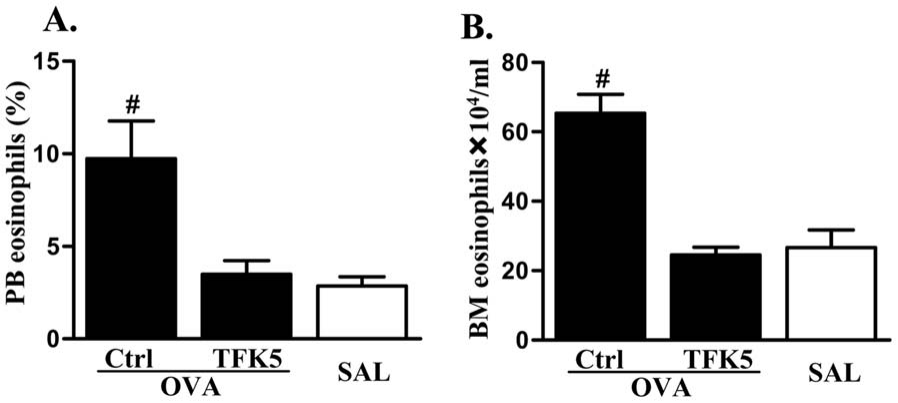
The effects of neutralization of IL-5 on eosinophil levels in peripheral blood and bone marrow in the absence of STAT6. The increased (A) percentages of eosinophils in peripheral blood and (B) numbers of eosinophils in bone marrow in response to OVA/OVA treatment were completely abolished by neutralization of IL-5 with anti-IL-5 antibody in STAT6-deficient mice. #P<0.05 compared to other groups.

We then assessed whether the accumulation of eosinophils in blood and development in bone marrow in the absence of STAT6 was associated with the STAT6-independent production of IL-5. OVA/OVA treated STAT6-deficient mice were administered IL-5 neutralizing mAb or isotype control every four days from day 22 d of the OVA/OVA treatment regime. Anti-IL-5 mAb treatment completely abolished the increases in the percentage of eosinophils in the peripheral blood of OVA/OVA treated STAT6-deficient mice (Figure 4A). The hematopoietic expansion of eosinophils in bone marrow in OVA/OVA treated STAT6-deficient mice was also completely abolished by neutralization of IL-5 (Figure 4B). Thus, STAT6 independent IL-5 production mediates eosinophil accumulation in the blood and development in bone marrow.

### CD4^+^ cells and NK cells cooperatively regulate the STAT6-independent development of eosinophils in bone marrow

**Figure 5:**
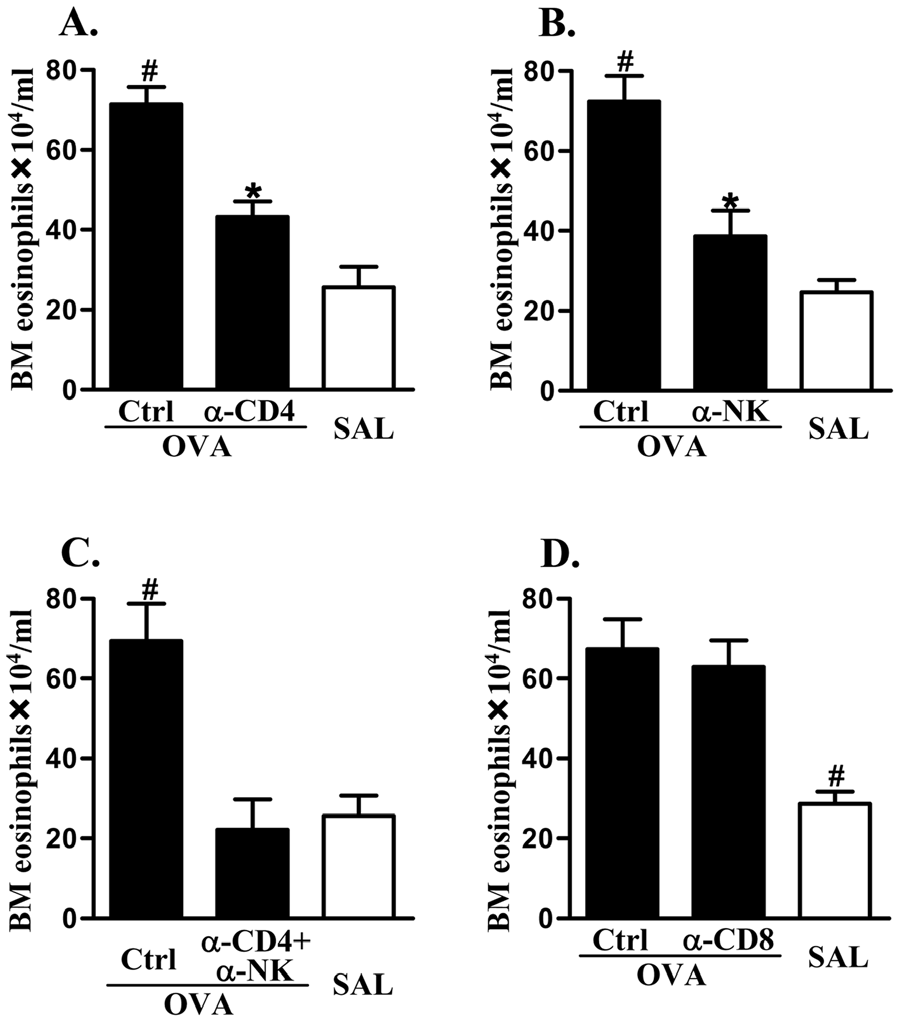
The effects of depletion of CD4^+^ and CD8^+^ T cells and NK cells on the development of eosinophils in the bone marrow in the absence of STAT6. (A) Anti-CD4 and (B) anti-NK cell (anti-ASIALO GM1) antibody significantly suppressed the increased development of eosinophils in the bone marrow of OVA/OVA treated STAT6-deficient mice. Combined treatment with (C) anti-CD4 and anti-NK antibodies completely abolished the increased development of eosinophils. By contrast, (D) anti-CD8 antibody had no effect. #P<0.05 compared to other groups. *P<0.05 compared to SAL/OVA treated mice.

CD4^+^- and CD8^+^-T cells and NK cells produce IL-5 and may regulate the expansion of eosinophils in bone marrow. Therefore, we then assessed the contribution of these cells on the STAT6-independent development of eosinophils. OVA/OVA treated STAT6-deficient mice were administered CD4^+^, NK, CD4^+^/NK or CD8^+^ cell neutralizing antibodies or isotype control every four days from day 22 d of the OVA/OVA treatment regime. Some studies have suggested that anti-ASIALO GM1 antibody may target other cells (e.g. CD8^+^ T cells or NK cells) [39], [40], however, many other investigations have found that this antibody specifically neutralizes NK cells without affecting other cells [41]–[43]. We have assessed the specificity and efficacy of NK cell depletion in our models. This was achieved by examining the levels of NK cells in the spleens of animals treated with anti-ASIALO GM1 antibody one day after the last OVA aero-challenge. The frequency of NK cells was 0.3±0.25% (mean ± SEM) compared with 4.1±0.5% (mean ± SEM) in isotype-treated animals. By contrast, the frequency of NKT cells, CD4^+^ and CD8^+^ T cells remained unchanged. This indicates that NK cells, but not NKT or T cell populations, were specifically depleted [41]–[43]. Depletion of CD4^+^ cells and NK cells significantly reduced the numbers of eosinophils in the bone marrow of OVA/OVA treated STAT6-deficient mice, however, numbers were still greater than in SAL/OVA treated controls (Figure 5A and B). However, importantly, combined treatment of antibodies against CD4 and NK cells completely abolished the increase in the numbers of eosinophil in the bone marrow of OVA/OVA treated STAT6-deficient mice, which were similar to the levels in SAL/OVA controls (Figure 5C). Furthermore, the increase in IL-5 was abolished by the combined administration of anti-CD4 and anti-NK cell antibodies (5.6±0.6 pg/ml, data expressed as mean ± SEM, n = 6, P<0.05), compared to isotype control (59±0.8 pg/ml), anti-CD4 (32.7±0.6 pg/ml) or anti-NK cell (34.3±0.4 pg/ml) antibodies alone. Indeed the levels were reduced to those in SAL/OVA treated WT (6.0±1.3 pg/ml) and STAT6-deficient mice (5.9±0.4 pg/ml). Administration of anti-CD8 antibody had no effect on eosinophil numbers (Figure 5D). These data suggest that STAT6-independent IL-5 production from CD4^+^ T cells and NK cells contributes to the development of eosinophil responses in allergic airway inflammation.

## Discussion

Here we demonstrate that STAT6 is a critical mediator of eosinophil influx into the lung. However, we also demonstrate that an alternative pathway that is independent of STAT6 controls the movement of eosinophils into the blood and partially mediates their development in the bone marrow during allergic airway inflammation. These effects were associated with reduced but still elevated levels of IL-5 in serum and the inhibition of the expression of eotaxins in the lung. We also show that IL-5 production, most likely from CD4^+^ and NK cells, may mediate STAT6-independent eosinophil accumulation in the blood and development in the bone marrow.

Other studies by Kuperman *et al*., using STAT6-deficient mice on a BALB/c background showed only a 50% reduction in eosinophil influx into the BALF during allergic airway inflammation [22]. These results are in direct contrast to our own and those of others that used STAT6-deficient mice on C57BL/6 or B6/129 backgrounds, which had >90% reduction in eosinophil influx [23], [24]. The differences of reduction of eosinophil influx observed by Kuperman *et al*., may be due to the differences in sensitization/challenge regime or mouse strain. By profiling gene transcripts of lung tissue from mice with two forms of experimental asthma (e.g. OVA- and *Aspergillus*-induced models), Zimmermann *et al*., found that a large number of genes are uniquely expressed independently of STAT6, including chemokines, membrane receptors, transcriptional regulators and enzymes [44]. The authors deduced that the regulation of some genes may be associated with, but are not necessarily restricted by STAT6 signalling. This study clearly indicates that alternative STAT6-independent signalling pathways exist that may contribute to asthma pathogenesis.

In our study, STAT6 deficiency led to reduced levels of eosinophils in bone marrow (Figure 2B) and IL-5 in serum (Figure 3A) in response to OVA/OVA treatment. Anti-IL-5 treatment abrogated eosinophil differentiation in bone marrow in the absence of STAT6 (Figure 4B). These results indicate that STAT6 deficiency contributes to, but is not essential for IL-5-induced eosinophil differentiation in the bone marrow. Since STAT6 is a critical mediator of Th2 responses [17], and therefore also IL-5 release, it may seem contradictory that eosinophil and IL-5 responses were detected in the blood and bone marrow in STAT6-deficient mice with allergic airway inflammation. This may result from compensatory mechanisms. Interestingly, others have shown that although IL-5 is not increased in response to allergen treatment in STAT6-deficient mice it is expressed at basal levels in the BALF of mice with a B6/129 background [24]. It is known that the activation of other signalling systems such as Notch/GATA3 or IL-2R/STAT5 pathways may also induce Th2 polarization and thereby IL-5 production, although in a less effective manner [45]–[47]. It is possible that it was the activation of these pathways that resulted in the STAT6-independent production of IL-5 and eosinophil responses and development in our study. The STAT6-independent production of IL-5 was responsible for the eosinophil development and movement into the blood since these responses were completely abolished by neutralization of IL-5. These results are in agreement with previous studies that i.v. injection of IL-5 (but not eotaxin) promotes the movement of eosinophils from the bone marrow into the circulation [7], [33]. These results confirm the critical role of IL-5 in the mobilization of the bone marrow pool of eosinophils into the blood.

Characterization of mRNA levels of chemokines in the lung revealed that the expression of eotaxin-1 and -2 were exclusively dependent on STAT6. Other chemokines, including MDC, TECK, TARC, RANTES, MCPs and MIPs were expressed independently of STAT6 in response to OVA/OVA treatment (unpublished data). The absence of eotaxin-1 and -2 in the lung, however, may have prevented the trafficking of eosinophils from the blood into lung tissue, and resulted in greater numbers of eosinophils in the circulation. Interestingly, although IL-5 was still produced in the lung (although markedly decreased) of OVA/OVA treated STAT6-deficient mice, the levels of eosinophils in the lung were not increased. These results confirm the importance of the STAT6-dependent production of these eotaxins and IL-5, by contrast to other chemokine subfamilies, in the recruitment of eosinophils to the airways during allergic inflammation.

CD4^+^ Th2 cells are the predominant cellular sources of IL-5 [5]. However, other cells such as CD8^+^ T-cells, NK cells and mast cells also produce this cytokine [48]. The expression of IL-5 by different cell types is controlled by variety of pathways. These different cells and pathways may differentially contribute to the development of eosinophilic responses in different tissue compartments during allergic reactions, which may depend on the timing and context of inflammatory stimuli and the immune microenvironment. NK cells produce IL-5 in allergic asthma [9], [49]. Interestingly, these cells do not produce IL-5 without IL-4 [49], suggesting that they need similar initiating signals, to those which promote Th2 immune responses. CD4^+^ NKT cells may also have a critical role in the pathogenesis of asthma and produce IL-5 when co-cultured with CD1d^+^ antigen presenting cells and IL-2 [50], [51]. Thus, both of these cells may contribute to IL-5 expression during acute inflammatory responses and there is evidence that crosstalk occurs between NK and NKT cells [52].

The depletion of CD4^+^ cells and NK cells in the absence of STAT6 inhibited eosinophilic responses in the bone marrow and the production of IL-5. These results suggest that although STAT6 pathways play a central role in the induction of eosinophilic responses, other pathways involving CD4^+^ T cells and NK cells may also contribute by independently producing IL-5 and inducing the development of eosinophilic responses in blood and bone marrow. Indeed increased numbers of NK cells (CD3^−^CD49b^+^FcεRII^−^) were detected in the bone marrow of OVA/OVA treated STAT6-deficient mice (unpublished data). These results indicate that a cooperative mechanism of STAT6-independent production of IL-5 may exist in CD4^+^ cells and NK cells and highlights the potential differential regulation of eosinophilic responses that is mediated by the surrounding inflammatory environment.

In summary, eosinophilic responses in allergic airway inflammation are differentially regulated by the activation of STAT6-dependent and -independent pathways at different parts of the inflammatory process. STAT6-dependent pathways critically regulate eosinophil migration into the lung, that involves the induction of eotaxin-1 and -2 expression. However, STAT6-independent IL-5-regulated eosinophilic pathways operate in blood and bone marrow compartments. These alternative pathways may be regulated by CD4^+^ and NK cells which produce IL-5 and contribute to the development of eosinophils in the bone marrow and their subsequent release into the blood. Therefore, therapeutic strategies that inhibit eosinophilia in eosinophil-mediated diseases should consider both pathways, even though both pathways are critically dependent on IL-5.
